# Tonsillar Trauma During Video Laryngoscopy: A Case Report

**DOI:** 10.7759/cureus.41617

**Published:** 2023-07-10

**Authors:** Bilal Tufail, Muhammad Shabbir, Amer Majeed, Ahmed Bilal Akhtar, Mohamed Al Malyan

**Affiliations:** 1 Anesthesia and Critical Care, King Faisal Specialist Hospital and Research Centre, Riyadh, SAU

**Keywords:** tonsils, trauma, difficult airway, injury, glidescope, video laryngoscopy, intubation

## Abstract

Potentially difficult airways warrant the use of airway adjuncts, which, if not used with caution, can cause trauma to the oral cavity. Although most operators are familiar with modern airway adjuncts, as they are not routinely used, adverse events can occur. Since its introduction, a video laryngoscope (VL) has been lauded as a necessary instrument for airway management in and out of the operating room. This case report highlights right tonsillar tissue perforation with a GlideScope® VL (Verathon Incorporated, Bothell, Washington, USA), requiring primary closure by an otolaryngologist.

## Introduction

A difficult airway can be defined as when an experienced provider anticipates or encounters difficulty with any or all facemask ventilation, direct or indirect laryngoscopy, tracheal intubation, supra-glottic device use, or surgical airway [1]. The use of a video laryngoscope (VL) has been on the rise, with positive results, especially in cases of difficult airways. In fact, in many hospitals, including ours, anesthesiologists prefer using VL as the most common tool to achieve a definitive airway in potentially difficult airway patients. VL as a modality of airway instrumentation has the potential to facilitate an unobstructed view of the vocal cords in situations where the oral, pharyngeal, and laryngeal axes are difficult to align, However, the Macintosh blade (Medsource®, Chanhassen, Minnesota, USA) is still the most widely used tool for intubating straightforward patients [2]. We report a case in which right tonsillar tissue perforation occurred due to the use of VL and required primary closure by an otolaryngologist.

## Case presentation

**Figure 1:**
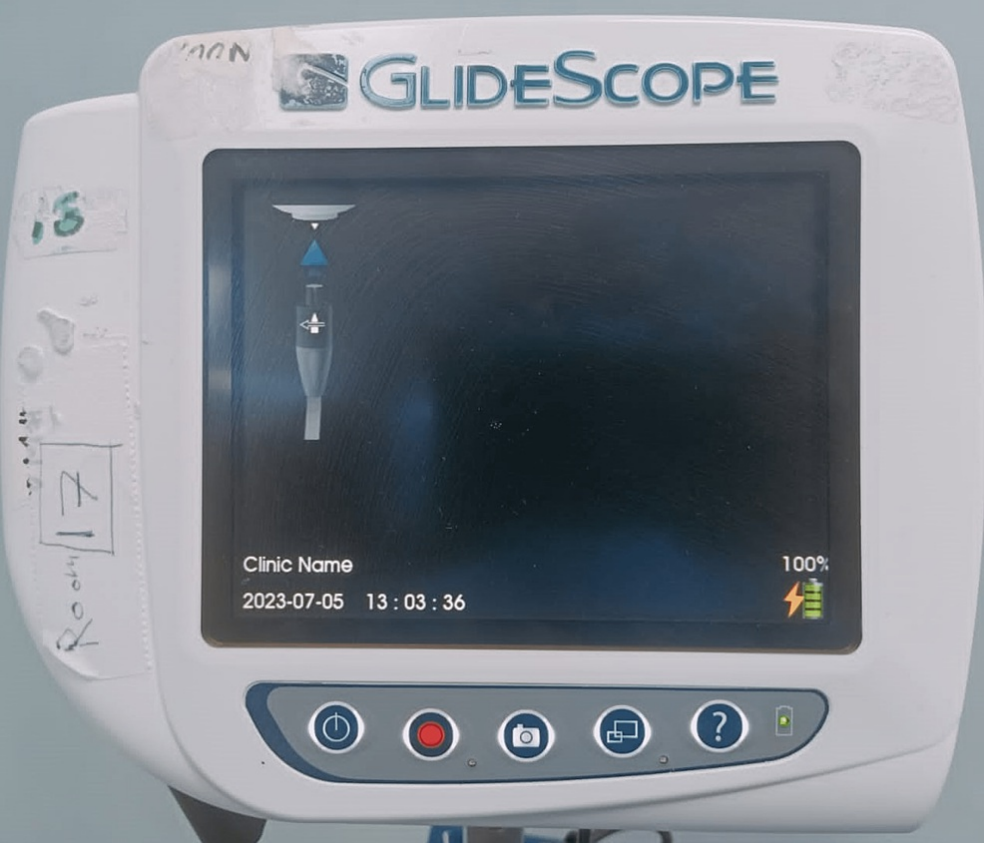
GlideScope® VL screen

**Figure 2:**
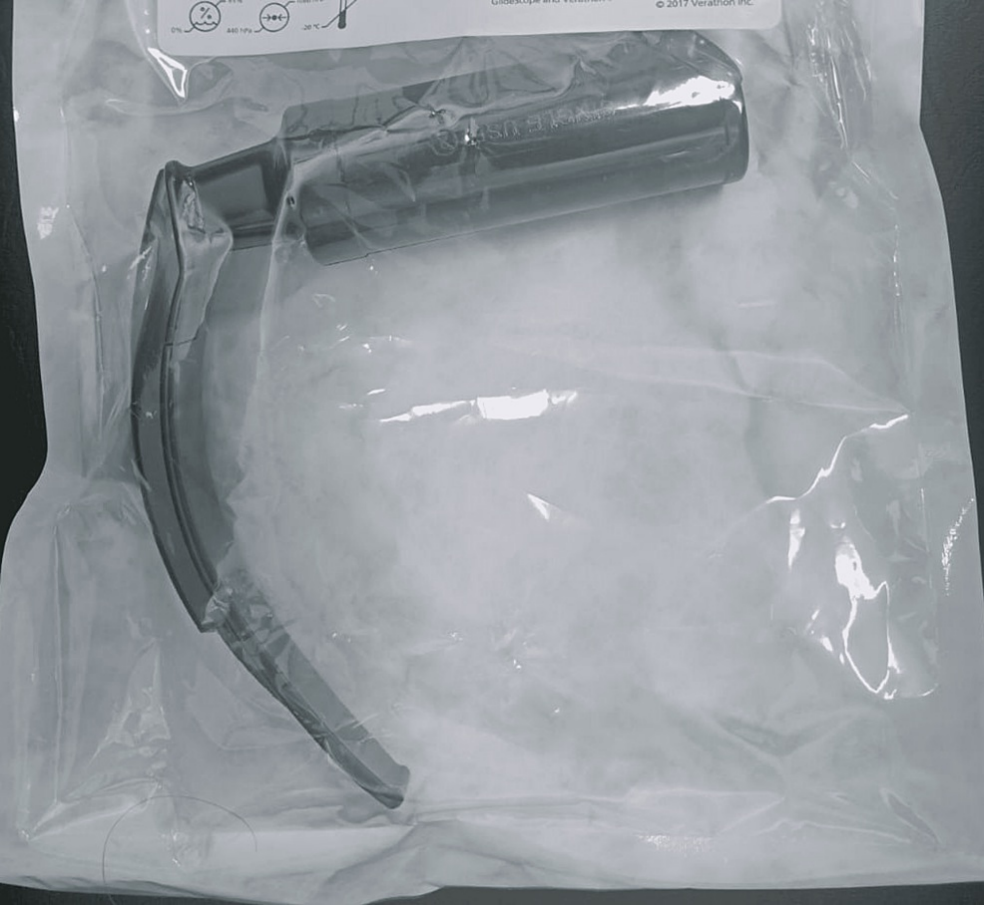
GlideScope® VL blade

**Figure 3:**
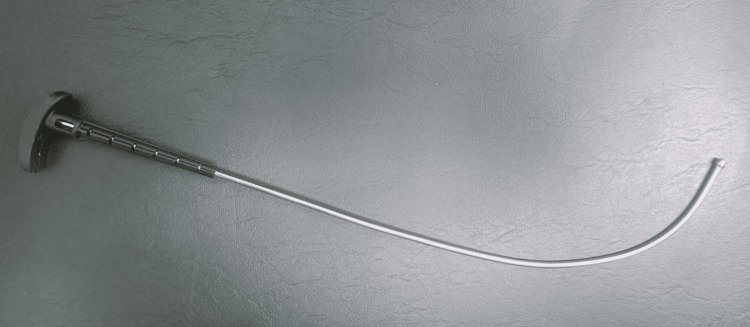
GlideScope® VL stylet

A 47-year-old male with Class II (severe) obesity according to the WHO classification (BMI 35 kg/m2), a known case of end-stage renal disease with Mallampati classification IV (only the hard palate is visible), was scheduled for living-related kidney transplantation. The rest of the airway examination results were unremarkable. Keeping in mind the above factors, we decided to intubate the patient with a GlideScope® VL (Figure 1-3). A difficult airway trolley, already organized according to plans A, B, C, and D, as per the Difficult Airway Society (DAS) guidelines, was available. After applying standard monitoring, including heart rate, non-invasive blood pressure (NIBP), saturation, and electrocardiography, the patient was preoxygenated in a ramped-up position for 3 min.

Induction of general anesthesia was initiated with a conventional dose of fentanyl (2 mcg/kg) and propofol (2 mg/kg). After the confirmation of bag-mask ventilation, atracurium (0.5 mg/kg) was given, and tracheal intubation was attempted using an 8 mm outer diameter cuffed endotracheal tube (ETT) ShileyTM Hi-Lo oral/nasal tracheal tube (Medtronic, Mansfield, Massachusetts, USA) with a GlideScope®. Despite adequate laryngoscopy, the ETT could not be passed on the first attempt. In the second attempt, the tube was angulated more with the GlideScope® stylet (Figure 2); however, this was still unsuccessful. It could only be passed successfully through the vocal cords on the third attempt with a 90-degree rightward rotation. Tracheal intubation was confirmed by visibly appreciating fogging within the ETT, auscultating bilateral breath sounds, and end-tidal carbon dioxide monitoring. No excessive force was applied during the laryngoscopy.

At the end of the procedure, oropharyngeal suctioning before tracheal extubation returned a significant volume of fresh blood. Examination of the oral cavity with a direct laryngoscope (DL), Macintosh blade size 3 (Medsource®, Chanhassen, Minnesota, USA), showed right tonsillar capsule perforation. Macintosh was used because the VL was larger and could have caused more trauma. Packing the right tonsillar pillar with adrenaline-soaked gauze did not control the bleeding. Hence, the otolaryngologist secured hemostasis by cauterizing and applying sutures to the lesions. The patient was extubated uneventfully after observation for half an hour. Follow-up on the 10th day confirmed that the laceration had healed without any residual damage.

## Discussion

VL has become popular as a user-friendly tool for the management of difficult intubation. In a Cochrane systematic review, VL was shown to have a lower rate of failed intubation than DL in adult patients, especially those with expected difficult airways [2]. In addition, the incidence of postoperative complications, such as upper airway trauma and hoarseness, was lower in VL.

However, various publications have reported numerous upper airway injuries using different types of VLs [3-5]. Greer et al. reported a statistically higher rate of injury using VL than DL [3]. The usual mechanism of injury is an outright soft tissue perforation. Tonsillar pillars and soft palate are the most common sites of trauma-related tissue injury secondary to laryngoscopy for intubation [3].

Soft tissue injuries commonly occur when the intubating physician concentrates on the video monitor and blindly inserts the GlideScope® into the oropharynx [4]. VL with an angulated blade, such as the GlideScope®, requires the use of a curved stylet to pass the ETT through the laryngeal opening [6]. The use of rigid stylets, such as the GlideRite® (Verathon Inc., Bothell, Washington, USA), significantly increases the risk of damage to upper airway structures; therefore, ETTs loaded with malleable stylets can reduce the rates of tissue injuries. Another study reported that the dedicated GlideScope®-specific rigid stylet (GRS) and standard malleable ETT stylet are equally effective in facilitating endotracheal intubation [7].

Advancing the ETT into the patient’s oral cavity and looking at the VL display creates a “blind spot” in the oropharynx, where the ETT cannot be seen until it is imaged in the camera’s view. It is recommended that the VL and ETT be inserted under direct vision before looking at the monitor display to avoid blind spots. Van Zundert suggested looking at the monitor screen after passing the ETT beyond the uvula [8].

In our case, the injury could have been due to repetitive laryngoscopy attempts, as the GlideScope® VL is very pointy and blunt. However, no bleeding was observed while the VL was in the oral cavity. One possibility is that the injury could have occurred while the VL was being removed from the mouth.

## Conclusions

VL may present an additional risk of airway trauma with instrumentation in blind spots, which are otherwise visible during DL. Furthermore, objects are heavy, pointed, and blunt, which can easily cause trauma without the application of excessive force. Therefore, attention should be paid to mitigating this risk when dealing with a potentially difficult airway, especially while removing the VL blade.
